# A molybdenum-promoted nickel–aluminum alloy catalyst for high-efficient hydrogenation reduction of nitrate to ammonia and nitrogen

**DOI:** 10.1039/d6ra02155g

**Published:** 2026-07-02

**Authors:** Ling-Feng Zou, Zi-Sheng Chao, Tao Xiang, Fen Wu, An Li

**Affiliations:** a College of Civil Engineering and Environment, Changsha University of Science and Technology Changsha 410082 China; b College of Materials Science and Engineering, Changsha University of Science and Technology Changsha Hunan 410114 China zschao@yahoo.com; c School of Chemistry and Chemical Engineering, Hunan Institute of Science and Technology Yueyang Hunan 414006 China anleechn@hotmail.com

## Abstract

Nitrate pollution in water necessitates efficient catalytic solutions for its conversion into harmless or valuable products. In this work, a molybdenum-promoted nickel–aluminum alloy (NAMCat) catalyst *via* alkaline leaching for the hydrogenation reduction of nitrate to N_2_ and NH_3_ was developed. The catalyst achieves over 99% nitrate conversion with brilliant product selectivity of N_2_/NH_3_ under mild conditions. The characterization results revealed that Mo doping critically induces a sponge-like porous architecture and fosters synergistic Ni–Mo active sites, which collectively enhance mass transport, hydrogen activation and reaction kinetics. The catalyst also demonstrates remarkable stability, recyclability, and tolerance to common interfering ions. A reaction mechanism based on the porous and electronic structure of NAMCat was also proposed. This work provides a cost-effective, non-noble-metal catalyst strategy for simultaneous nitrate remediation and nitrogen resource recovery.

## Abstract 2

A molybdenum-promoted Ni–Al alloy catalyst was developed for highly-efficient nitrate hydrogenation to N_2_/NH_3_. It achieved >99% conversion with remarkable stability, recyclability, and tolerance to common interfering ions.

## Graphical abstract

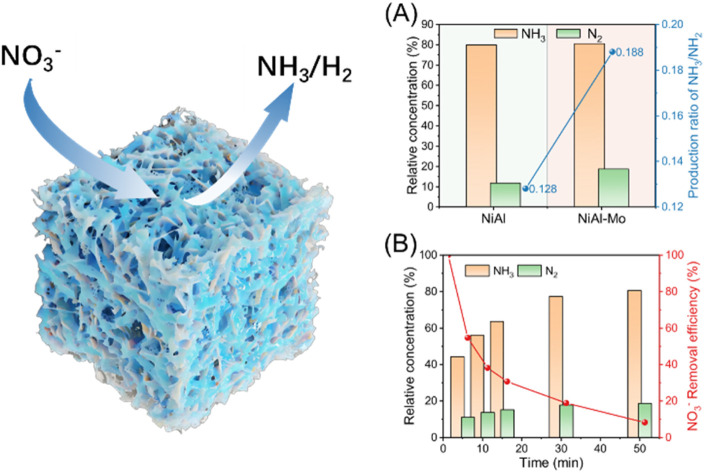

## 1 Introduction

Nitrate (NO_3_^−^) pollution represents one of the most pressing challenges for the global aquatic environment, primarily stemming from the excessive use of agricultural fertilizers, industrial wastewater discharge, and domestic sewage. Its accumulation in water bodies not only leads to eutrophication but also poses a direct threat to human health through its conversion into nitrite and nitrosamines.^1–3^ Therefore, the development of efficient, economical, and environmentally friendly technologies for deep nitrate removal has become an urgent need in the fields of environmental catalysis and water resource reuse.^4,5^

Current methods for nitrate-containing wastewater treatment primarily include biological denitrification,^6,7^ ion exchange,^8–10^ electrochemical reduction,^11–13^ photoelectrocatalytic reduction^14,15^ and hydrogenation reduction,^16–18^ aiming for efficient and low-carbon nitrate removal. Among those nitrate removal technologies, catalytic hydrogenation reduction is considered one of the most promising methods, due to its advantages of fast reaction rates, absence of secondary pollution, and the direct conversion of nitrate into harmless nitrogen (N_2_) or valuable ammonia (NH_3_).^16–18^ The core of this technology lies in the design and preparation of high-performance catalysts. Early research predominantly focused on noble metal-based catalysts (*e.g.*, Pd–Cu, Pt–Sn), which despite demonstrating high activity, face limitations for widespread application due to their high cost and resource scarcity_3_.^16–21^ Consequently, developing efficient catalysts based on inexpensive non-precious metals has become key to advancing the practical application of this technology.^22–24^

Among non-precious metals, nickel (Ni) has attracted significant attention due to its abundant reserves, low cost, and good hydrogen activation capability.^25,26^ considering single Ni-based catalysts often encounter issues such as agglomeration of active sites, insufficient stability, bimetallic alloy catalysts have been widely constructed by introducing a second metallic component, utilizing the synergistic effects to modulate catalytic performance. In particular, Ni–Al based alloy catalysts can not only enhance Ni dispersion and prevent sintering of the active component through the introduction of aluminum but also improve the intrinsic activity of Ni by modulating its electronic structure.^27–30^ For instance, Prof. of F. Devred Team used Ni–Al catalyst for high selective hydrogenation of citral, *via* optimizing the microstructure derived from residual aluminum in the bulk phase, achieved through controlling the NiAl_3_ phase content in the alloy and employing atomization preparation technology.^27^

In recent years, the introduction of a third component (*e.g.*, Mo, Fe, Co) into the Ni–Al alloy catalysts for multi-metal doping to further optimize catalyst performance has become a research focus.^31–33^ The doping of Mo facilitates the formation of intermetallic compounds like Ni–Mo, enhances the hydrogen evolution reaction activity of the catalyst, and may improve hydrogenation efficiency by promoting water dissociation. Iron (Fe), as a low-cost and environmentally friendly promoter, can induce electronic structure reconstruction in Ni-based catalysts through doping, optimizing the hydrogen adsorption energy, thereby enhancing hydrogen activation capability and reaction kinetics. For example, a ternary NiCoFe alloy achieved a near-optimal hydrogen adsorption free energy (−0.12 eV) by lowering the d-band center of Ni to −2.74 eV through charge redistribution, significantly boosting catalytic activity.^34^ Thus, the introduction of a third component can modulate the d-electron structure of nickel, weakening the over-adsorption of reaction intermediates, which is expected to enhance catalytic activity for the hydrogenation reduction of nitrate to N_2_/NH_3_.

In this paper, the molybdenum-doping Ni–Al based alloy catalyst was prepared, and further employed for catalytic hydrogenation reduction of nitrate to generate NH_3_ and N_2_. The catalytic activity and influencing factors were systematically investigated, and the structures of the catalyst were characterized *via* XRD, SEM and XPS, further revealing the structure–activity relationship between catalysts structure and catalytic performance. By constructing an efficient, stable and low-cost Ni–Al based multi-component alloy catalytic system, this work aims to provide new material design concepts and a theoretical basis for nitrate pollution control and water resource reuse.

## 2 Experimental

### 2.1. Materials

All the chemicals, including sodium hydroxide, nitrate, ammonium hydroxide, Nessler's reagent, *N*-(1-naphthyl)-ethylenediamine and so on, had analytic purity and purchased from Shanghai Aladdin Biochemical Technology Co., Ltd. The commercially nickel–aluminum (weight ratio of Ni : Al = 50 : 50) and molybdenum-doped nickel–aluminum (weight ratio of Ni : Al : Mo = 49 : 49 : 2%) alloy powder were supplied by JiaHong Chemical Co., Ltd.

### 2.2. Catalysts preparation

**Fig. 1:**
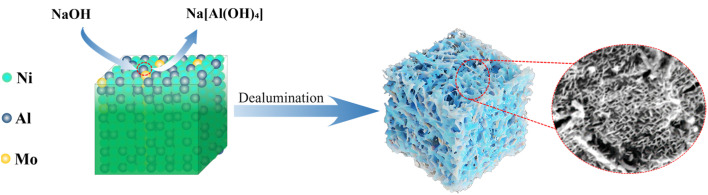
The diagram of preparation process for the NAMCat catalyst.

The alloy-based catalysts were activated through selective leaching in an alkaline solution (Fig. 1). The specific activation procedure was conducted according to the previous literatures.^27^ Firstly, a predetermined mass of sodium hydroxide (NaOH) solid was weighed and dissolved in an deionized water to obtain an 20% concentration of alkaline solution. Subsequently, the molybdenum-doped nickel–aluminum alloy powder was slowly added to the alkaline solution at 50 °C under continuous stirring. Then, the obtained suspension was kept stirring at 50 °C for 2 hours, in order to adequately activate catalyst *via* aluminum dissolution. After the reaction, the supernatant alkaline solution was then carefully decanted, and the solid product was subsequently washed alternately with deionized water and absolute ethanol for several cycles until the pH of the final wash effluent became neutral. Finally, the resulting catalyst was stored in absolute ethanol, namely NAMCat.

As comparison, the parent nickel–aluminum alloy powder was also used as raw materials to prepare nickel–aluminum alloy catalyst as the above operating steps, the obtained catalyst was named as NACat.

### 2.3. Catalysts characterization

The X-ray diffraction (XRD) measurement for synthesized catalysts was performed on a Bruker D8 diffractometer using Cu Kα radiation with a wavelength of 1.54187 Å. The scanning electron microscopy (SEM) image was acquired on a JEOL JSM 6700 F field emission scanning electron microscope at an accelerating voltage of 10 kV. The X-ray photoelectron spectroscopy (XPS) analysis was performed using a PHI Quantum 2000 microscope equipped with Al Kα radiation source. A suitable amount of catalyst was compressed into a wafer for analysis.

### 2.4. Catalytic hydrogen reduction of nitrate

Catalytic hydrogen reduction of nitrate ions was performed in the batch reaction apparatus (Fig. 1S). The batch operation for the catalytic reduction of nitrate (NO_3_^−^–N) proceeded as follows: (1) the measured reaction solution was precisely added to the batch reactor. (2) The solution in the reactor was purged with H_2_ for 30 minutes to remove dissolved oxygen. (3) The catalyst was added to the reactor to initiate the reaction. The sample with the volume of 1 mL was collected from the reactor at specific time intervals, and filtered through a 0.22 µm membrane filter. In the cycle test of catalyst, the catalyst was collected by centrifugation, washed with deionized water repeatedly after each run, and reused directly under identical conditions.

The filtration was analyzed using a UV-Vis spectrophotometer to determine the concentrations of NO_3_^−^, NO_2_^−^, and NH_4_^+^. The concentration of NO_3_^−^–N was determined using the ultraviolet spectrophotometric method, according to the existence significant absorption of NO_3_^−^ ions at the wavelength of 220 nm, which allows for the measurement of NO_3_^−^–N concentration in water. The concentration of NO_2_^−^–N was analyzed by the *N*-(1-naphthyl)-ethylenediamine spectrophotometric method. The concentration of NH_4_^+^–N was determined using the Nessler's reagent spectrophotometric method. The degradation of NO_3_^−^–N was calculated *via* Formula (1-1), and the yield of NO_2_^−^–N, NH_4_^+^–N and N_2_–N was calculated *via* Formula (1-2)–(1-4), respectively.1-1
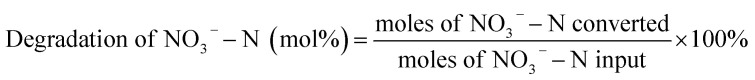
1-2
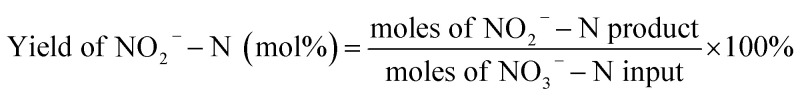
1-3
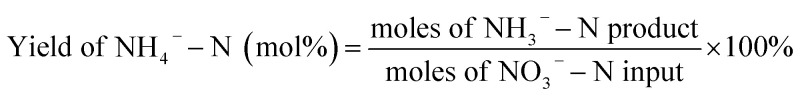
Moles of N_2_–N product = [degradation of NO_3_^−^–N] − [yield of (NO_2_^−^–N + NH_4_^−^–N)]1-4
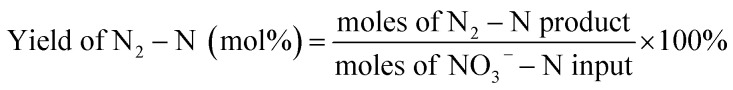


## 3 Result and discussion

### 3.1. Catalysts performances

**Fig. 2:**
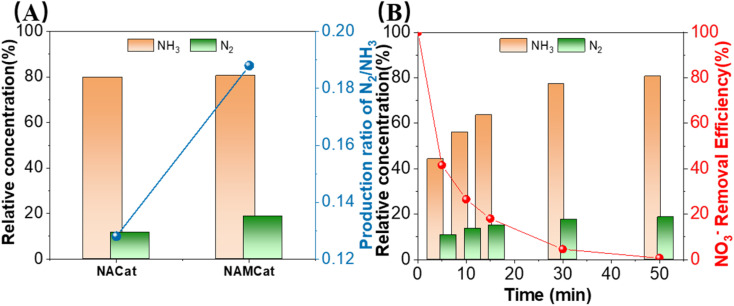
The performance of NACat and NAMCat catalysts for degradation of nitrate. (A) The relative concentration of NH_3_ (yellow, bar), N_2_ (green, bar), and the production ration of N_2_/NH_3_ (blue, line) after a 50 min reaction of each sample were determined. (B) The hydrogenation reduction performance of NAMCat at the reaction time from 5 to 50 min. Reaction condition: initial nitrate–nitrogen (NO_3_^−^–N) concentration: 100 mg L^−1^, reaction time: 50 min, reaction temperature: 25 °C, H_2_ flow rate: 90 mL min^−1^.

Fig. 2 illustrates the performance of nickel–aluminum alloy catalyst (NACat) and molybdenum-promoted nickel–aluminum alloy catalyst (NAMCat) catalyst for degradation of nitrate. Both of catalysts exhibited distinct catalytic properties over a 50 minute reaction period (Fig. 2A). The NACat catalyst achieved a nitrate conversion of 91.8%, while the NAMCat catalyst demonstrated better performance with the nitrate conversion increasing to 99.3%. Furthermore, the ratio of N_2_/NH_3_ reached up to 0.188 over the NAMCat catalyst, which was significantly higher than that of the NACat catalyst (0.128). The result indicated that the NAMCat catalyst possessed the better catalyst activity and selectivity for N_2._ In a 50 min time processing measurement over NAMCat catalyst (Fig. 2B), the NO_3_^−^ removal efficiency achieved to 99% based on the relative NO_3_^−^ concentration as time processing. When the reaction time reached 50 min, the yield of NH_3_ is 80.6% and N_2_ is 18.7%, respectively. Notably, a small accumulation of the NO_2_^−^ intermediate was observed during the initial reaction stage (5–15 minutes) and gradually eliminated as the reaction proceeded, indicating the NAMCat catalyst exhibited good capability for subsequent processing of reaction intermediates.

**Fig. 3:**
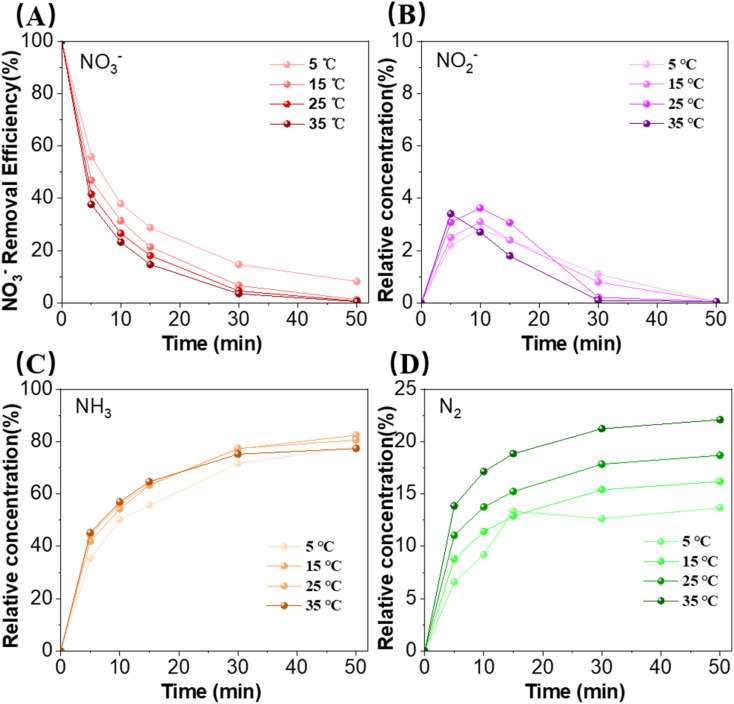
The reaction temperatures for the degradation performance of nitrate over NAMCat catalysts. (A) NO_3_^−^ removal efficiency, (B) relative concentration of NO_2_^−^, (C) relative concentration of NH_3_, (D) relative concentration of N_2_.

Fig. 3 presents the catalytic performance for hydrogenation reduction of nitrate over the NAMCat catalyst at different temperatures. After a 50 minute reaction, the NO_3_^−^ conversion reached 91.8% at 5 °C, and further increased with increasing temperature. As a result, the NO_3_^−^ conversion could reached 99.5% At 35 °C. Meanwhile, nitrite (NO_2_^−^) remained at a consistently low level (≤1.0%) throughout the process, indicating its rapid conversion as a reaction intermediate. Meanwhile, the ammonia (NH_3_) yield showed initially increasing and subsequent decreasing trend with increasing temperature, reaching maximum value to 82.5% at 15 °C. In contrast, the nitrogen (N_2_) yield increased obviously, rising from 13.7% at 5 °C to 22.1% at 35 °C. Prior studies reveal that the hydrogenation of nitrate occurs through a sequential reduction process, involving the transition from NO_3_^−^ to NO_2_^−^, then to reactive nitrogen intermediates, and finally to gaseous N_2_ or NH_3_.^20,35,36^ Thus, the low NO_2_^−^ concentration suggests its subsequently fast reaction. Meanwhile, the collision frequency between active nitrogen intermediates and hydrogen species could be increased, which promotes the continuous hydrogenation pathway toward main product of NH_3_. In comparison, the N_2_ formation pathway requires the coupling of two nitrogen-containing intermediates on the catalyst surface, resulting in a more limited increase in N_2_ yield. Thus, the results indicate that the NAMCat catalyst exhibits strong nitrate degradation activity under relatively low temperatures.

**Fig. 4:**
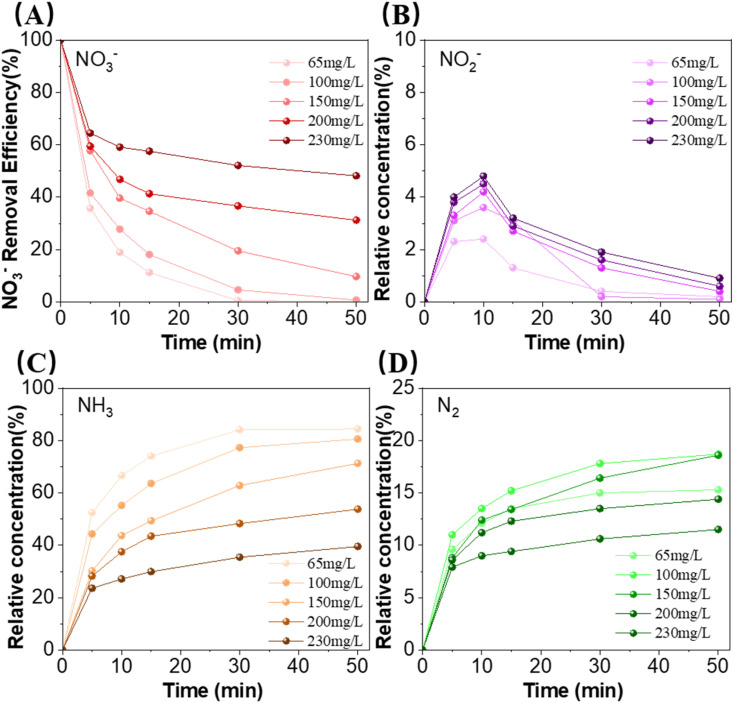
The initial concentration for the degradation performance of nitrate over NAMCat catalysts. (A) NO_3_^−^ removal efficiency, (B) relative concentration of NO_2_^−^, (C) relative concentration of NH_3_, (D) relative concentration of N_2_.

Fig. 4 illustrates the effect of initial nitrate concentration on the degradation performance. As the initial concentration increased from 65 mg L^−1^ to 230 mg L^−1^, the NO_3_^−^ conversion decreases obviously. The NO_3_^−^ conversion dropped from 99.8% at 65 mg L^−1^ to 51.9% at 230 mg L^−1^ after 50 minutes. Simultaneously, the NH_3_ yield systematically decreased from 84.4% to 39.5%. In contrast, the N_2_ yield initially increased and then declined with the increasing of initial nitrate concentration, arriving to maximum value with 18.7% at 100 mg L^−1^. This behavior is probably attributed to saturation of surface active site at high concentrations. The NO_3_^−^ conversion was hindered at very high concentrations, due to mass transfer limitations.

**Fig. 5:**
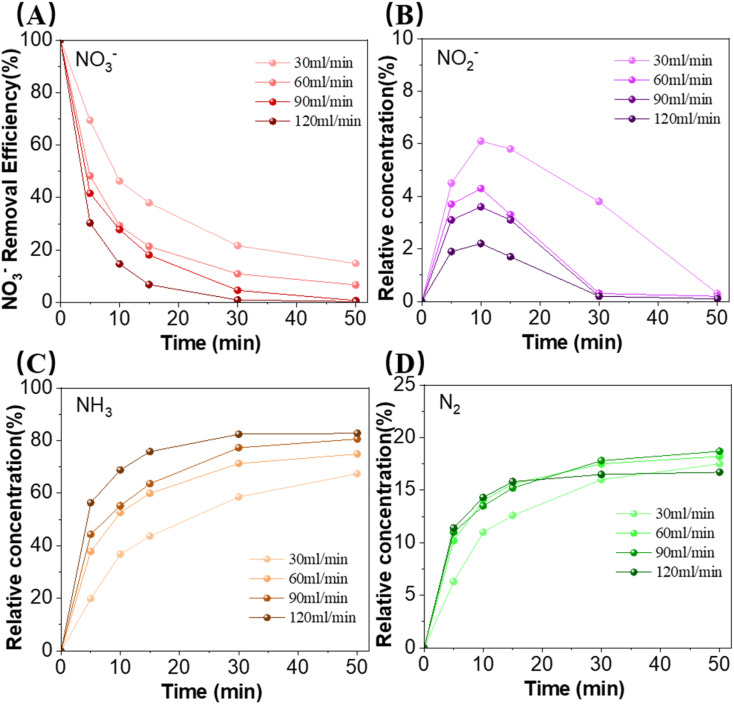
The flow of H_2_ for the degradation performance of nitrate over NAMCat catalysts. (A) The NO_3_^−^ removal efficiency, (B) the relative concentration of NH_3_, (C) the relative concentration of NO_2_^−^, and (D) N_2_ produced were shown in color red, yellow, green and blue circle scatters.

Fig. 5 illustrates the effect of hydrogen flow rate on nitrate degradation performance. The NO_3_^−^ conversion increases observably with higher hydrogen flow. After 50 minutes, the NO_3_^−^ conversion rose from 85.2% at 30 mL min^−1^ to 99.7% at 120 mL min^−1^, and the NH_3_ yield increased steadily from 67.4% to 82.8%. While the N_2_ yield initially rises and the decreases, reaching to maximum yield of 18.7% at 90 mL min^−1^. Meanwhile, the accumulation of NO_2_^−^ intermediate was significantly reduced from 6.1% at 30 mL min^−1^ to below 2.2% at 120 mL min^−1^. The observed effects are attributed to enhanced gas–liquid mass transfer. Higher hydrogen flow improved the renewal efficiency at the gas–liquid interface, increasing the supply of dissolved hydrogen to the active sites of catalyst. Besides, the Mo–Ni synergy in the catalyst promoted efficient hydrogen dissociation,^37,38^ creating a hydrogen-rich microenvironment,^39,40^ which favors the deep hydrogenation pathway to NH_3_.

**Fig. 6:**
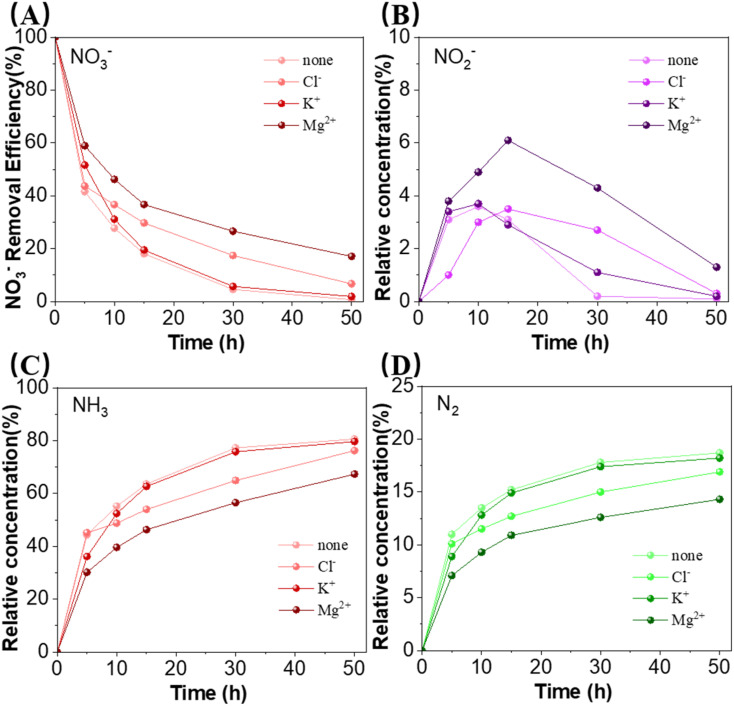
The interfering ion for the degradation performance of nitrate over NAMCat catalysts. (A) NO_3_^−^ removal efficiency, (B) relative concentration of NO_2_^−^, (C) relative concentration of NH_3_, (D) relative concentration of N_2_.

Fig. 6 illustrates the effect of interfering ions on nitrate degradation performance. The presence of K^+^, Cl^−^, and Mg^2+^ ions differentially influenced the catalytic process through distinct ionic interaction mechanisms. The highest NO_3_^−^ conversion after 50 minutes was achieved in the interfering ions-free system. This was slightly reduced to 98.1% in the presence of K^+^, significantly lowered to 93.3% with Cl^−^, while strongly suppressed to 82.9% with Mg^2+^. In K^+^ interfering system, the relatively high activity for yield of NH_3_ and N_2_ was maintained well. In Cl^−^ interfering system, the NH_3_ yield in the Cl^−^ system was 76.2% closest to that of the interfering ions-free control, while its N_2_ yield decreases to 16.9%. However, In Mg^2+^ interfering system, the catalytic activity exhibited the strongest suppression, significantly reducing NH_3_ and N_2_ yields to 67.3% and 14.3% respectively. The results are attributed to specific ion–surface interactions. K^+^ likely compresses the electrical double layer, moderately affecting mass transfer. Cl^−^ can specifically adsorb onto active sites of catalyst, poisoning and altering electronic structure, which probably particularly suppresses the coupling pathway to generate N_2_. Mg^2+^, prone to hydrolysis and precipitation as Mg(OH)_2_ under alkaline conditions, can physically cover active sites and block pores, leading to severe inhibition and intermediate accumulation.^41,42^

**Fig. 7:**
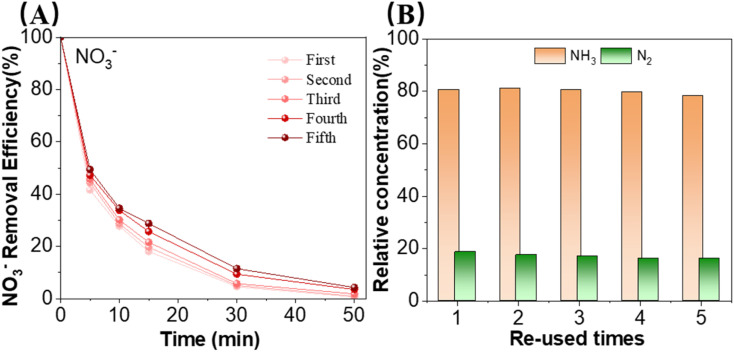
The recycling performance of NAMCat catalyst for the degradation of nitrate. (A) The NO_3_^−^ removal efficiency, and (B) the relative concentration of NH_3_ (yellow, bar), N_2_ (green, bar).

Fig. 7 demonstrates the effect of catalyst recycling on nitrate degradation performance. After five recycling uses, the catalyst maintained a high 95% NO_3_^−^ conversion over a 50 minute reaction period, representing only a slight decrease from the first use (99.3%). The NH_3_ yield remained well stable across the five cycles. In contrast, the N_2_ yield showed a gradual decline from 18.7% to 16.2%. As a result, the NAMCat catalyst shows the excellent stability for NO_3_^−^ degradation performance.

### 3.2. Catalysts characterization

**Fig. 8:**
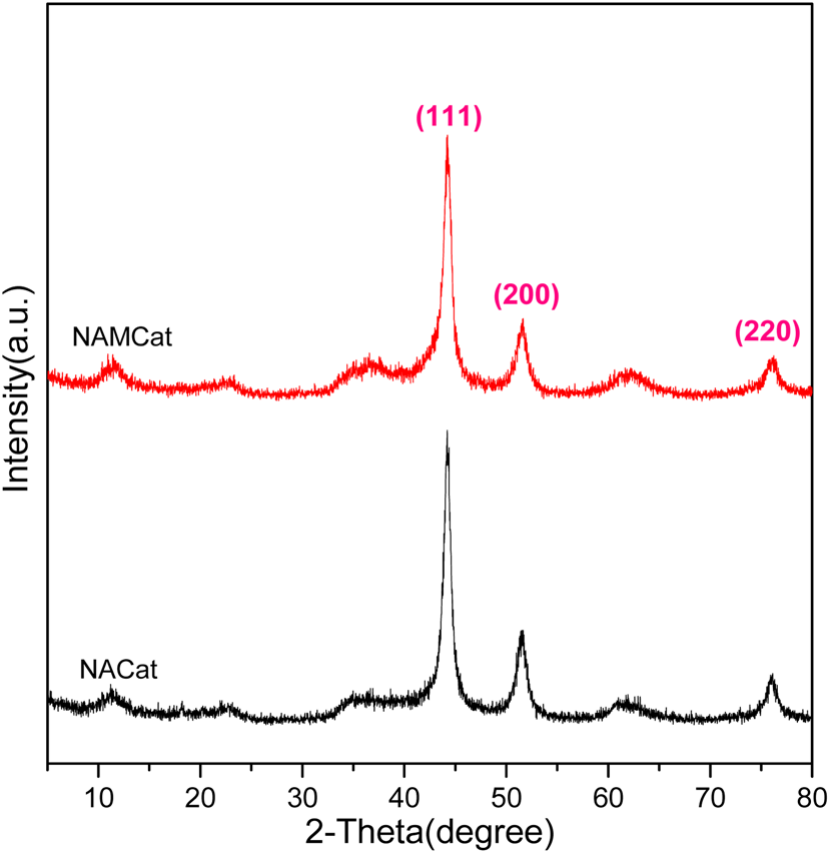
The XRD patterns for the NACat and NAMCat catalysts.

Fig. 8 presents the X-ray diffraction (XRD) patterns of the NACat and NAMCat catalysts. For both catalysts, strong characteristic diffraction peaks were observed at 44.5°, 51.8°, and 76.4°. These peaks were identified as the (111), (200), and (220) planes of face-centered cubic (fcc) metallic nickel (Ni^0^) by comparison with the standard card (JCPDS no. 04-0850).^43,44^ Notably, no diffraction peaks corresponding to nickel-aluminide intermetallic compounds (*e.g.*, Ni_3_Al, NiAl) were detected.^45,46^ This suggests that aluminum atoms did not form long-range ordered intermetallics but entered the fcc-Ni lattice as a substitutional solid solution Ni(Al). Meanwhile, no distinct diffraction peaks for molybdenum oxides or molybdenum–nickel compounds were detected in the NAMCat catalyst. This indicates that Mo atoms were highly dispersed, likely forming an atomic-level solid solution within the fcc-Ni(Al) lattice.^47,48^ A slight broadening of the main fcc-Ni(Al) (111) peak was observed for the NAMCat catalyst compared to the NACat catalyst. According to the Scherrer equation of *D* = *Kλ*/(*β* cos *θ*), the increase of full width at half maximum (*β*) leads to the decrease of crystallite size. This result indicates a reduction in crystallite size of NACat catalyst, which may result from the lattice microstrain and/or reduction in crystallite size induced by the incorporation of Mo atoms into the Ni lattice, forming a highly dispersed solid solution.^47^

**Fig. 9:**
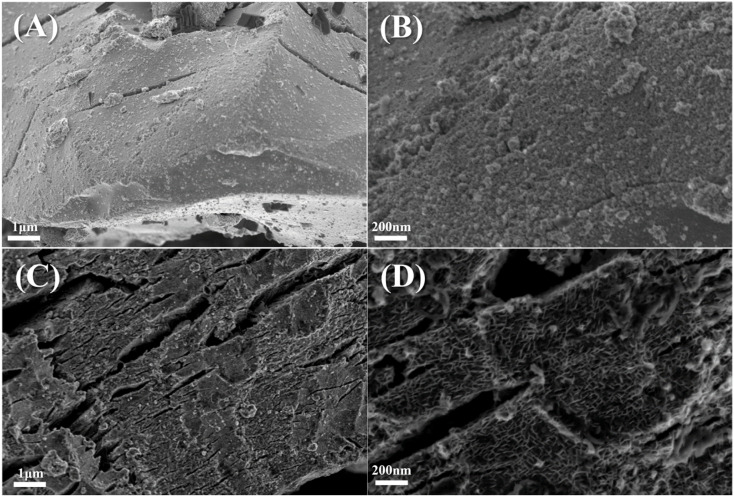
The SEM for the NACat and NAMCat catalysts. (A) and (B) correspond to NACat; (C) and (D) correspond to NAMCat.

Fig. 9 presents scanning electron microscopy (SEM) images of the NACat and NAMCat catalysts. The undoped NACat catalyst exhibited a relatively dense and smooth overall morphology (Fig. 9A and B). The compact surface with sporadic shallow pits and cracks for NACat catalyst indicated that aluminum leaching likely occurred primarily at the surface and grain boundaries, without forming an extensive, interconnected porous network. In contrast, the microstructure of the Mo-doped NAMCat catalyst was markedly different (Fig. 9C and D). A sponge-like porous structure with a three-dimensional (3D) network of interconnected macropores was formed, indicating that the abundant mesopores and interparticle gaps was created. This significant morphological difference is probably attributed to the effect of the dopant element.^49–51^ Molybdenum likely introduces microstructural heterogeneities that act as preferential pathways, enabling deeper and more uniform penetration of the alkaline etchant and efficient, selective dissolution of aluminum. The resulting developed porous structure provides a high surface area, exposes more active nickel sites, and ensures efficient mass transport, which is crucial for catalytic performance.^51–53^ Furthermore, the morphology and microstructure of the catalysts were also characterized by HR-TEM (Fig. 2S). Both samples exhibit aggregated nanosheet-like structures with no obvious large metal particle agglomeration (Fig. 2SA and C). Meanwhile, the well-defined lattice planes further indicate that Mo introduction does not disrupt the host crystal structure (Fig. 2SB and D). These results demonstrate that the catalysts possess well-maintained crystallinity and good active species dispersion, both favorable for catalytic performance. Besides, the N_2_ adsorption–desorption isotherms indicate that the NACat and NAMCat catalysts exist mesopore (Fig. 2S), and the NAMCat catalyst possesses larger specific surface area, pore volume and mesopore diameter than the NAMCat one (Table 1S), which facilitate mass transport and expose more active sites.

**Fig. 10:**
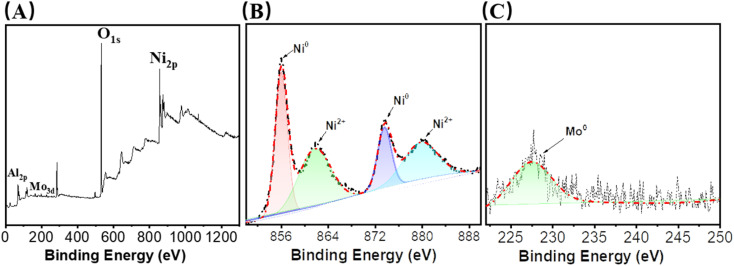
The XPS patterns for the NAMCat catalyst. (A) XPS spectra of NAMCat, (B) Ni_2p_ spectra of NAMCatand (C) Mo_3d_ spectra of NAMCat.

Fig. 10 presents the X-ray photoelectron spectroscopy (XPS) patterns of the NAMCat catalyst. The survey spectrum clearly shows characteristic photoelectron peaks for Ni, Al, Mo and O. The relative intensities indicate that Ni is the dominant metal surface element, while the Mo signal is weak due to its low doping level. The Ni_2p_ spectrum was divided into the 2p_3/2_ and 2p_1/2_, due to the coexistence of metallic Ni^0^ and Ni^2+^ species (Fig. 10B). The predominant peaks at around 855.5 eV and 872.6 eV were attributed to metallic Ni^0^ (2p_3/2_ and 2p_1/2_, respectively), revealing that metallic Ni^0^ is the predominant surface nickel species, which serves as the key active site for hydrogen dissociation. Meanwhile, the weaker peaks at approximately 860.2 eV and 879.5 eV are ascribed to Ni^2+^ in NiO (2p_3/2_ and 2p_1/2_), which was due to surface oxidation of metallic Ni upon air exposure. Besides, the low-intensity Mo_3d_ spectrum could be deconvoluted at approximately 257.8 eV, indicating the presence of Mo species.^54^ These highly dispersed Mo species may modulate the electronic structure, such as the d-band electron density of adjacent Ni atoms, potentially optimizing reactant adsorption and enhancing catalytic activity.^55–57^

In summary, the molybdenum-promoted nickel–aluminum alloy (NAMCat) catalyst possessed a highly porous sponge-like structure, synergistic Mo–Ni bimetallic active sites, and a surface hydrophilic alumina layer. This unique architecture facilitates efficient mass transport, enhances hydrogen activation, and optimizes reactant adsorption, collectively contributing to its superior catalytic performance in nitrate hydrogenation.

### 3.3. Reaction mechanism of nitrate hydrogenation reduction

**Fig. 11:**
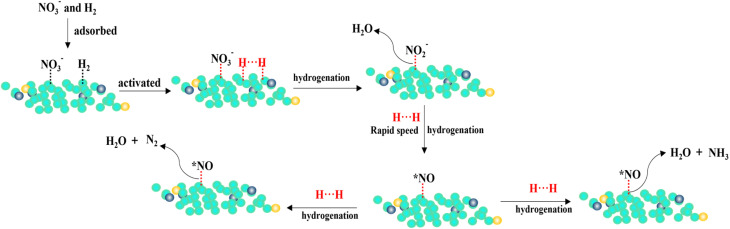
The possible reaction mechanism of nitrate hydrogenation reduction over NAMCat catalyst.

For the hydrogenation reduction reaction of nitrate over NAMCat catalyst, a synergistic reaction mechanism for efficient nitrate hydrogenation is proposed (Fig. 11), according to the structure and composition of molybdenum-promoted nickel–aluminum alloy catalyst. Initially, the nitrate ions (NO_3_^−^) were adsorbed onto the catalyst surface of NAMCat. The sponge-like porous structure provides a high surface area and enhances mass transport, ensuring efficient reactant access.^58^ Concurrently, hydrogen molecules (H_2_) are readily activated and dissociated on the metallic Ni^0^ sites, act as the predominant active hydrogen. Through the synergistic Ni–Mo bimetallic sites, the highly dispersed Mo species could modulate the electronic structure of adjacent Ni atoms, likely optimizing the adsorption energy of key intermediates activity.^55–57^ Subsequently, the NO_3_^−^ was reduced by active hydrogen to NO_2_^−^ and further to generate active nitrogen intermediates, such as *NO and *NH_*x*_.^59^ Finally, these intermediates on the hydrogen-rich surface affected the selectivity of nitrogen/ammonia. The deep hydrogenation process leads to ammonia formation, while the coupling of two nitrogen-containing intermediates yields nitrogen.^22^ Both of porous structure of catalyst and synergy effect of Ni–Mo bimetallic sites affected the microenvironment of hydrogenation reduction, and then regulate relative rates of these competing steps, achieving high conversion and tunable selectivity.

## 4 Conclusion

In this paper, a high-performance molybdenum-promoted nickel–aluminum alloy (NAMCat) catalyst was successfully developed *via* an alkaline leaching process for efficient hydrogenation reduction of nitrate to nitrogen and ammonia. The catalyst demonstrates exceptional catalytic activity, achieving over 99% nitrate conversion with enhanced nitrogen selectivity under optimized conditions. The results of comprehensive characterizations reveal that Mo doping plays a crucial role in forming a unique sponge-like porous structure and creating synergistic Ni–Mo active sites, which collectively improve mass transport, hydrogen activation and reaction kinetics. As a result, the catalyst exhibits brilliant catalytic activity, remarkable stability and tolerance to interfering ions. Besides, a synergistic reaction mechanism for efficient nitrate hydrogenation was also proposed. However, the catalytic performance is inhibited at high nitrate concentrations, and the detailed structure evolution of the catalyst during cycling has not been fully revealed. Future work will focus on optimizing the catalyst structure, studying the deactivation mechanism and exploring practical wastewater treatment. In summary, this work provides a cost-effective and sustainable strategy for simultaneous nitrate removal and resource recovery, exhibiting the potential of metal-doping alloy catalysts for environmental remediation.

## Conflicts of interest

The authors declare no conflicts of interest regarding this manuscript.

## Supplementary Material

RA-016-D6RA02155G-s001

## Data Availability

All raw experimental data, analysis data and relevant code supporting the findings of this study are available within the supplementary information (SI) of this article. Supplementary information is available. See DOI: https://doi.org/10.1039/d6ra02155g.
