# Supplementary material for: A molybdenum-promoted nickel–aluminum alloy catalyst for high-efficient hydrogenation reduction of nitrate to ammonia and nitrogen
Source: RSC Adv. 2026 Jul 2;16(34):32908–18. doi: 10.1039/d6ra02155g (PMC13326161; doi:10.1039/d6ra02155g)
Supplement: RA-016-D6RA02155G-s001 [file RA-016-D6RA02155G-s001.pdf]

Supplementary Information (SI) for RSC Advances.
This journal is © The Royal Society of Chemistry 2026

Supporting Materials

A molybdenum-promoted nickel-aluminum alloy catalyst for high-efficient hydrogenation reduction of Nitrate to Ammonia and Nitrogen

Ling-Feng Zou [1], Zi-Sheng Chao [*, 2], Tao Xiang [3], Fen Wu [3], An Li [*,3]

[1] *College of Civil Engineering and Environment, Changsha University of Science and Technology, Changsha, 410082, China*

[2] *College of Materials Science and Engineering, Changsha University of Science and Technology, Changsha, Hunan, 410114, China*

[3] *School of Chemistry and Chemical Engineering, Hunan Institute of Science and Technology, Yueyang, Hunan, 414006, China*

***Corresponding author.** Email: zschao@yahoo.com (Z.S. Chao); anleechn@hotmail.com (A. Li)

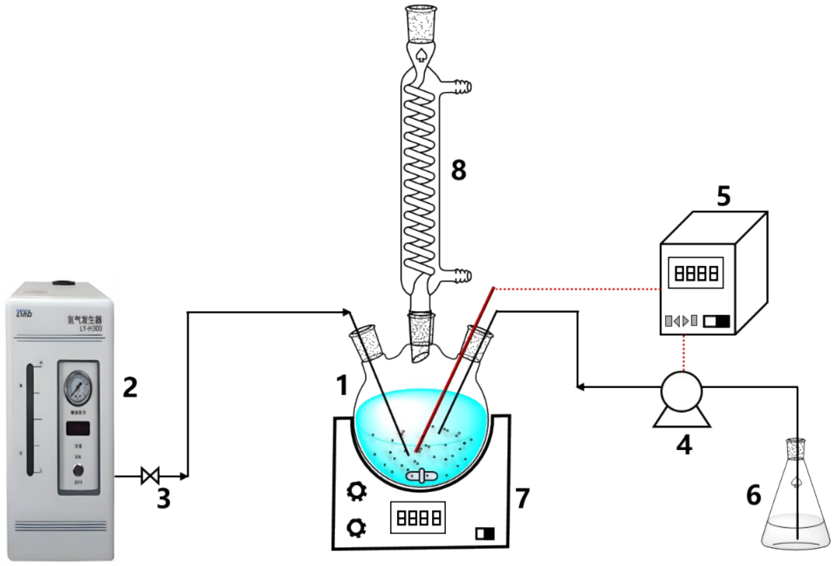

Figure 1S. the batch reaction apparatus of catalytic hydrogen reduction of nitrate

1. Reactor; 2. Hydrogen generator; 3. One-way valve; 4. Peristaltic pump; 5. pH meter; 6. Acid solution; 7. Temperature controller; 8. Condenser.

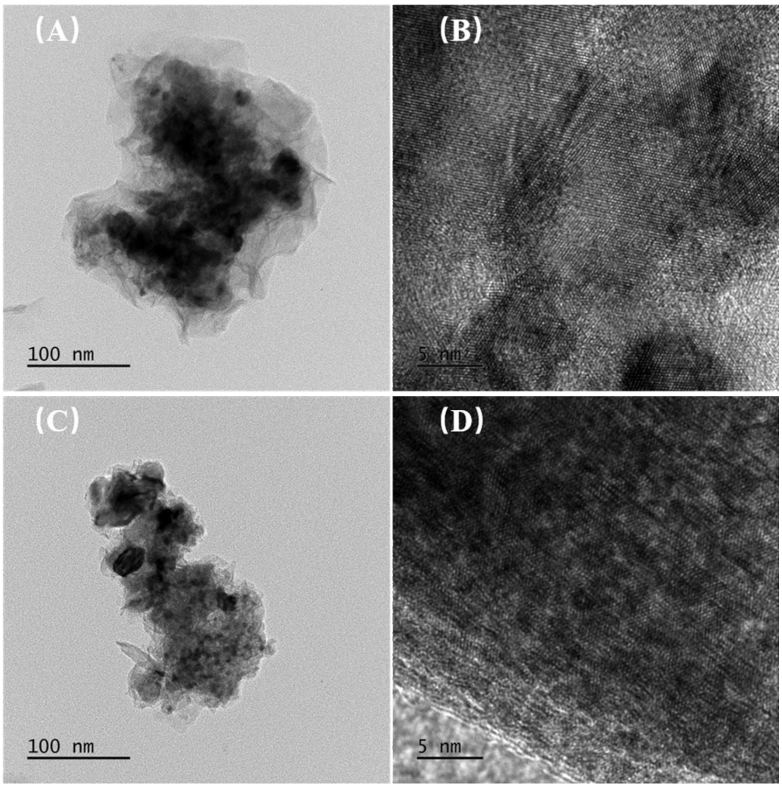

Figure 2S. the HR-TEM for the NACat and NAMCat catalysts. (A) and (B) correspond to NACat；(C) and (D) correspond to NAMCat

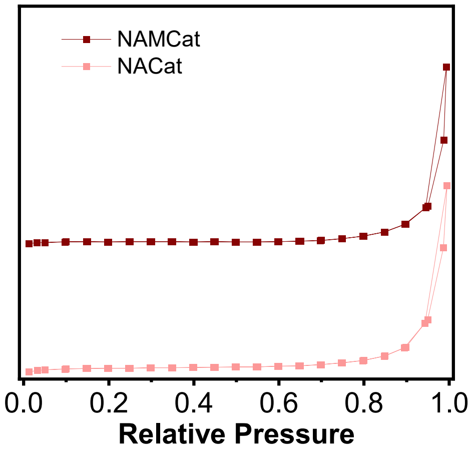

Figure 3S. The N_2 adsorption-desorption isotherms for the NACat and NAMCat catalysts.

Table 1S. the textural properties for the NACat and NAMCat catalysts.

Catalyst	S_{BET} (m^2/g)	V_{total} (cm^3/g)	D_{meso} (nm)
NACat	87.3	0.09	3.93
NAMCat	98.6	0.10	5.70

S_{BET} refers to specific surface area, Vt_{otal} refers to total pore volume. D_{meso} refers to the mesopore diameter calculated via BJH method.
